# Quantitative and pattern recognition analyses of magnoflorine, spinosin, 6′′′-feruloyl spinosin and jujuboside A by HPLC in Zizyphi Semen

**DOI:** 10.1007/s12272-013-0295-z

**Published:** 2013-12-06

**Authors:** Won Il Kim, Bing Tian Zhao, Hai Yan Zhang, Je Hyun Lee, Jong Keun Son, Mi Hee Woo

**Affiliations:** 1 College of Pharmacy, Catholic University of Daegu, Gyeongsan, 712-702 Korea; 2 College of Pharmacy, Yeungnam University, Gyeongsan, 712-749 Korea; 3 College of Oriental Medicine, Dongguk University, Gyeongju, 780-714 Korea

**Keywords:** 6′′′-Feruloyl spinosin, Jujuboside A, Magnoflorine, Spinosin, Zizyphi Semen

## Abstract

Two rapid and simple HPLC methods with UV detector to determine three main compounds (magnoflorine, spinosin and 6′′′-feruloyl spinosin) and evaporative light scattering detector (ELSD) to determine jujuboside A were developed for the chemical analyses of Zizyphi Semen. Magnoflorine, spinosin, and 6′′′-feruloyl spinosin were separated with an YMC J’sphere ODS-H80 column (250 mm × 4.6 mm, 4 μm) by the gradient elution followed by the isocratic elution using methanol with 0.1 % formic acid and water with 0.1 % formic acid as the mobile phase. The flow rate was 1.0 mL/min. Jujuboside A was separated by HPLC–ELSD with YoungJinBioChrom Aegispak C18-L column (250 mm × 4.6 mm, 5 μm) column in a gradient elution using methanol with 0.1 % formic acid (A) and water with 0.1 % formic acid as the mobile phase. These two methods were fully validated with respect to linearity, precision, accuracy, stability, and robustness. These HPLC methods were applied successfully to quantify four compounds in a Zizyphi Semen extract. The HPLC analytical methods were validated for pattern recognition analysis by repeated analysis of 91 seed samples corresponding to 48 *Zizyphus jujuba* var*. spinosa* (J01–J48) and 43 *Zizyphus mauritiana* (M01–M43). The results indicate that these methods are suitable for a quality evaluation of Zizyphi Semen.

## Introduction

Zizyphi Semen is the dried seeds of *Zizyphus jujuba* Miller var. *spinosa* Hu ex H. F. Chou (*Z. jujuba* var. *spinosa*) in the Korean Pharmacopoeia (K.P.) and the Chinese Pharmacopoeia (C.P.), and belongs to the Rhamnaceae family (Lee et al. 1996). It is distributed mainly in tropical and subtropical regions of the world. Zizyphi Semen has been used as an analgesic, a tranquilizer, and an anticonvulsant in oriental countries such as Korea and China for over 2,500 years (Han et al. 2009). It has been used as an anticonvulsant and for treating anxiety and insomnia in folk medicine in India (Pahuja et al. 2012), and for treating depression, insomnia, and anxiety in other Asian countries (Liu et al. 2012).

Studies have found that Zizyphi Semen possesses beneficial effects on the cardiovascular system such as anti-arrhythmia and anti-hypertension (Fu et al. 2011), anti-anxiety (Peng et al. 2000), amelioration of seizures and oxidative stress (Pahuja et al. 2011), enhancement of pentobarbital-induced sleep (Ma et al. 2008), protection of *N*-methyl-d-aspartate-induced neuronal cell damage (Park et al. 2004), inhibition of histamine release (Mao et al. 2007), reduction of atherosclerosis by inhibiting foam cell formation (Fujiwara et al. 2011) and prevention of food-borne pathogens (Al-Reza et al. 2010).

Magnoflorine, one of main alkaloid components in Zizyphi Semen (Lee et al. 2012), has anti-glycemic (Patel and Mishra 2012) and antioxidant (Rackova et al. 2004) activities. Spinosin, another major flavonoid compound (Lee et al. 2012), potentiates pentobarbital-induced sleep via a serotonergic mechanism (Wang et al. 2008, 2010). Jujuboside A, the other main component (Cheng et al. 2000), has been studied for its effect on hippocampal neurons of rat (You et al. 2010) and for insomnia (Wang et al. 2012).

The regulation of Zizyphi Semen content in C.P. (2010) has been already stipulated in 2010; it is prescribed to contain no less than 0.08 % spinosin and 0.03 % jujuboside A from *Z. jujuba* var. *spinosa*. However, the K.P. (2013) has no stipulation on the main compounds contained in Zizyphi Semen. The purpose of this study was to establish a reliable high-performance liquid chromatographic (HPLC) method to quantitatively analyze the major compounds in Zizyphi Semen, and to provide analytical method which would be used as the official analytical method in K.P. revision. The dried seeds of *Z. mauritiana,* which are normally distributed and cropped in low-latitudes of Asia, Africa, and Australia (Ji et al. 2012), is mislabeled as Zizyphi Semen in Korean herbal markets. Therefore, we also suggest analytical marker compounds to distinguish the seeds of *Z. jujuba* var. *spinosa* from those of *Z. mauritiana.*

In previous studies, several analytical methods such as ultraviolet spectrophotometry (Li and Li 2001), liquid chromatography/mass spectroscopy (Liu et al. 2007; Li et al. 2008), ultra-high-performance liquid chromatography coupled with diode-array detector (UPLC–DAD) (Niu and Zhang 2011) and HPLC–UV (Shin et al. 1982) have been established to quantify or identify the components in Zizyphi Semen. Ultraviolet spectrophotometry, targeting spinosin and other flavonoids, is a simple method but does not provide detailed chemical information like retention times of magnoflorine and jujuoboside A (Li and Li 2001). HPLC-photo diode array detection and HPLC–DAD–electrospray ionization–mass spectroscopy (HPLC–DAD–ESI–MS) method had been developed to identify 11 compounds including spinosin, 6′′′-feruloyl spinosin, and jujuboside A in Zizyphi Semen, in which a complicated elution method more than 6 steps and long running time (65 min) were used (Liu et al. 2007). UPLC–DAD has been applied for chromatographic fingerprint analysis and quantitative analysis of six flavonoids to classify and discriminate 23 Zizyphi Semen samples, but had complicated elution conditions like poor elution times of 15.45 or 22.95 min. HPLC chromatogram also exhibited some overlapped peaks of marker compounds (Niu and Zhang 2011). In C.P., two methods, such as HPLC–UV to determine spinosin and HPLC–ELSD to determine jujuboside A, have been used to assay marker compounds in Zyziphi Semen. However, complicated elution conditions were used for both methods. Magnoflorine, a major marker compound with different content between *Z. jujuba* var. *spinosa* and *Z. mauritiana* resulted from this study, was not adopted as a marker compound for Zyziphi Semen.

In this study, newly developed method not only has short analytical time but also shows good resolution. Magnoflorine, one of maker compounds, was not adopted in conventional experiments, even though it was regarded as an important marker compound in this study.

We suggest a suitable analytical method for quantitative and pattern recognition analyses of Zyziphi Semen together with the establishment of appropriate marker compounds to distinguish between *Z. jujuba* var. *spinosa* and *Z. mauritiana*.

## Materials and methods

### Reagents and materials

**Fig. 1:**
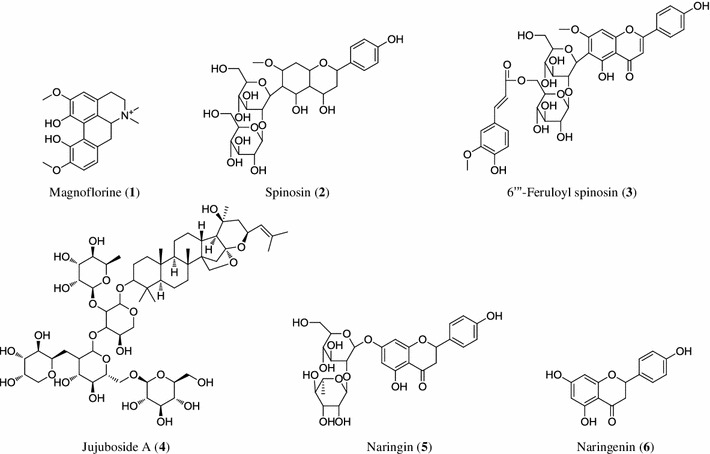
Structures of standards and an internal standards

The magnoflorine (**1**), spinosin (**2**), 6′′′-feruloyl spinosin (**3**), and jujuboside A (**4**) standards were kindly provided by the Zizyphi Semen separation team of Korean National Center for Standardization of Herbal Medicines, which were separated from *Z. jujuba* var. *spinosa.* The internal standards (I.S.), naringin (**5**) and nargingenin (**6**), were purchased from Sigma-Aldrich (St. Louis, MO, USA). The compound structures are shown in Fig. 1. The purities of these compounds were determined to be >98 % by normalizing the peak areas detected by HPLC analyses. Methanol was purchased from Merck K GaA (Darmstadt, Germany). All other chemicals used were analytical grade. Deionized water was prepared using the Milli-Q purification system (Millipore, Bedford, MA, USA). This study adopted the seed samples of 48 *Z. jujuba* var*. spinosa* (J01–J48), and 43 *Z. mauritiana* (M01–M43). All *Z. jujuba* var*. spinosa* samples (J01–J48) originated from China in the provinces of Hebei, Shaanxi, Shandong and Sichuan. The *Z. mauritiana* samples originated from China (M04, M06, M12–M14, M20, and M21), Vietnam (M18 and M26), and Myanmar (M01–M03, M05, M07–M09, M10, M11, M15–M17, M19, M22–M25, and M27–M43). All of these samples were provided by Prof. Je Hyun Lee (College of Oriental Medicine, Dongguk University, Gyeongju, Korea).

### Sample preparation

Each standard stock solution was prepared by adding 1.0 mg magnoflorine, spinosin and 6′′′-feruloyl spinosin to 1.0 mL of methanol containing 80 ppm naringin, respectively. A standard stock solution was prepared by adding 1.0 mg jujuboside A to 1.0 mL of methanol containing 50 ppm naringenin.

A powdered sample of Zyziphi Semen (1.0 g) for HPLC–UV was mixed with 50 mL of 50 % methanol containing 80 ppm I.S. (naringin) in a vial and the mixture was refluxed for 30 min. A powdered sample of Zyziphi Semen (5.0 g) for HPLC–ELSD was mixed with 50 mL of 50 % methanol containing 50 ppm I.S. (naringenin) in a vial. Each mixture was sonicated for 30 min. The solution was weighed again, and the loss in weight was made up with methanol. The solution was filtered through a 0.45-μm membrane filter (Whatman), and the filtrate was used as the test solution. A 10 μL aliquot of the test solution was injected into the HPLC system.

### HPLC–UV conditions

The HPLC equipment was a Waters HPLC system (Empower pro) with a Waters 600 pump, a Waters 486 tunable absorbance detector and Waters 717 autosampler (Waters Inc., Milford, MA, USA). Three different columns were used and compared: YMC J’sphere ODS-H80 (250 mm × 4.6 mm, 4 μm), YoungJinBioChrom Aegispak C18-L (250 mm × 4.6 mm, 5 μm) and Phenomenex Gemini ODS C18 (250 mm × 4.6 mm, 5 μm). The mobile phase consisted of water containing 0.1 % formic acid (A) and methanol containing 0.1 % formic acid (B). Elution was performed at a flow rate of 1 mL/min in gradient and isocratic modes. The solvent gradient was changed according to the following program: from 90 % (A): 10 % (B) to 60 % (A): 40 % (B) at 0–10 min; and 60 % (A): 40 % (B) at 10–40 min. The column was washed by 100 % of (B) for 20 min and re-equilibrated by 90 % (A): 10 % (B) for 20 min. The mobile phase was filtered under vacuum through a 0.21-μm membrane filter and was degassed prior to use. Chromatograms were acquired at 270 nm by a UV detector.

### HPLC–ELSD conditions

The HPLC equipment was a Gilson HPLC system (Unipoint 2.0) with a Gilson 321 pump, a Gilson Prep TM II ELSD detector and Gilson 321 XL auto-sampler (Gilson Inc. Middleton, WI, USA). The above three different columns were compared in HPLC–ELSD and two mobile phases, A and B, were also same with HPLC–UV. Elution was performed at a flow rate of 0.1 mL/min in a gradient mode. The solvent gradient was changed according to the following program: from 45 % (A):55 % (B) to 25 % (A):75 % (B) at 0–30 min. The column was washed by 100 % of (B) for 20 min and re-equilibrated by 45 % (A):55 % (B) for 20 min. The mobile phase was filtered under vacuum through a 0.21-μm membrane filter and was degassed prior to use. The ELSD parameters of the spray chamber and drift tube temperatures, and gas pressure were optimized at 30, 60 °C and 50 psi, respectively.

### Analytical method validation

The developed HPLC method was validated according to Korea Food and Drug Administration (KFDA) guidelines for the following parameters: linearity, limits of detection (LOD), limits of quantification (LOQ), accuracy, precision, and robustness.

### Linearity

A standard stock solution was prepared and diluted to an appropriate concentration to construct the calibration curves. The calibration curve for HPLC–UV was composed of seven concentrations of 0.625, 6.25, 12.5, 25, 50, 100, and 200 μg/mL. The calibration curve was constructed by plotting the peak area ratio (magnoflorine/I.S., spinosin/I.S., 6′′′-feruloyl spinosin/I.S.) with seven different concentration values. The calibration curve for HPLC–ELSD was composed of six concentration levels of 25, 35, 50, 75, 100, and 200 μg/mL. The calibration curve was constructed by plotting the logarithm of the peak area ratio (jujuboside A/I.S.) with the logarithm of the six different concentration values.

### Limits of detection and quantification

The lowest concentration of working solution was diluted with appropriate concentrations, and LOD and LOQ under the chromatographic conditions were separately determined at signal-to-noise ratios (S/N) of about 3 and 10, respectively.

### Accuracy and precision

Precision and accuracy were determined in HPLC–UV by spiking three concentration levels of the magnoflorine, spinosin, and 6′′′-feruloyl spinosin standards, which were mixed with a Zyziphi Semen (J14) sample for subsequent extraction and filtration. Three concentrations of 0.9, 90.0, and 135.0 μg/mL for magnoflorine and spinosin, and 1.0, 100.0, and 150.0 μg/mL for 6′′′-feruloyl spinosin were evaluated. Precision and accuracy in HPLC–ELSD were determined as the same way except three concentrations of 40.0, 100.0, and 200.0 μg/mL were used with the jujuboside A standard. The HPLC–UV and HPLC–ELSD analytical experiments were performed in triplicate for each control level. Data from the standard solution and the extracted sample were compared. Precision and accuracy were determined by multiple analyses (*n* = 5) of quality control samples prepared at low, medium and high concentrations spanning the calibration range.

### Robustness

The robustness of the method was studied by introducing changes in the column (i.e., J’sphere, Aegispak, Gemini), column temperature (i.e., 25, 30, 35, and 40 °C) and flow rates (i.e., 0.8, 1.0, and 1.2 mL/min).

### Pattern recognition analysis

A pattern recognition analysis was conducted to evaluate the phytochemical equivalency among the 91 samples (48 *Z. jujuba* var. *spinosa* (J01–J48), 43 *Z. mauritiana* (M01–M43) samples). We used two major marker compound HPLC–UV peaks of magnoflorine and spinosin, and one major marker compound HPLC–ELSD peak of jujuboside A for the pattern recognition analysis using IBM SPSS Statistics Version 19 software (SPSS, Inc., Chicago, IL, USA).

## Results

### Optimization of chromatographic conditions

**Fig. 2:**
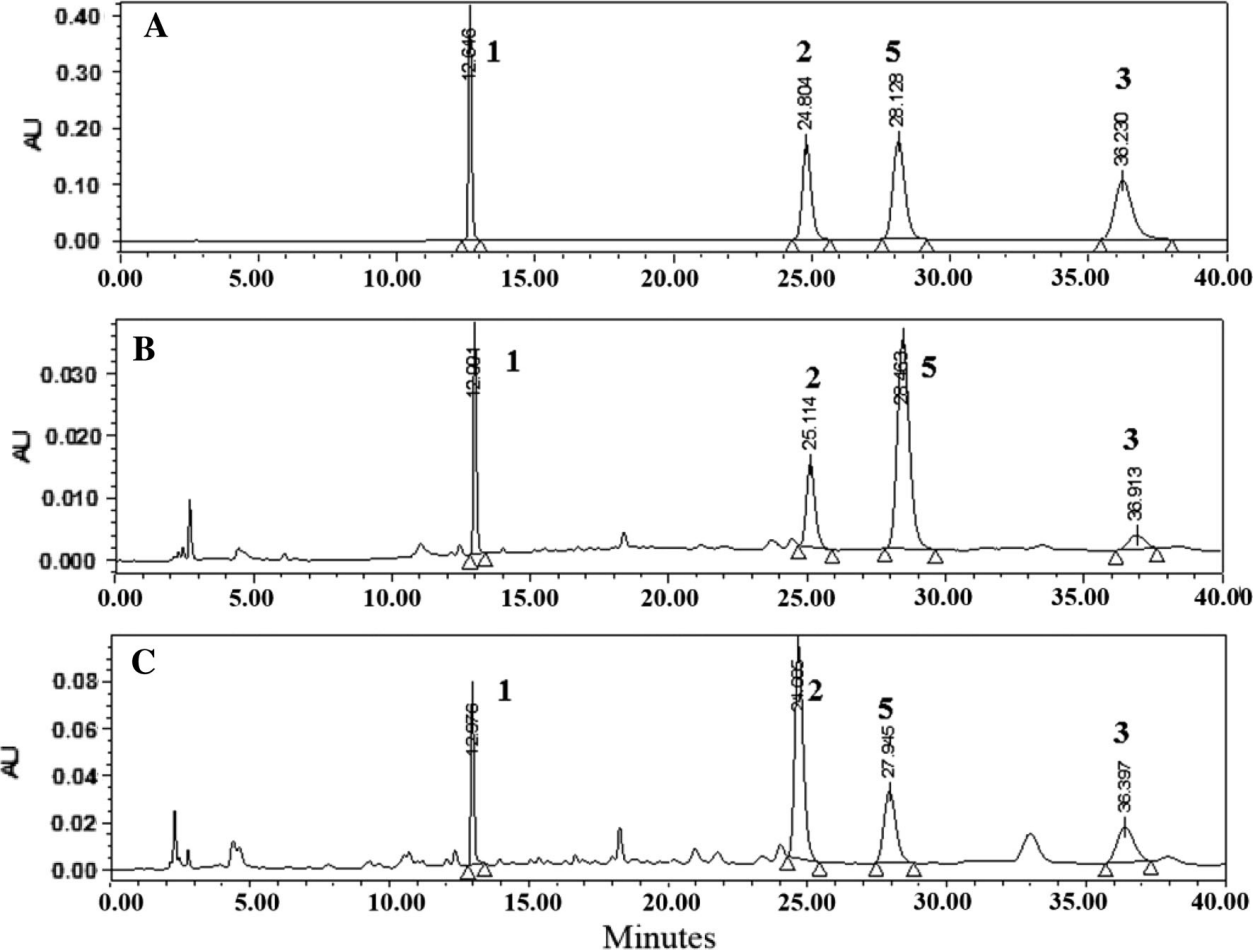
HPLC–UV chromatograms of standard mixture (**a**), the sample of *Z. jujuba* var*. spinosa* (J01, **b**) and the sample of *Z. mauritiana* (M01, **c**). *1* Magnoflorine, *2* Spinosin, *3* 6′′′-Feruloyl spinosin, *5* Naringin

**Fig. 3:**
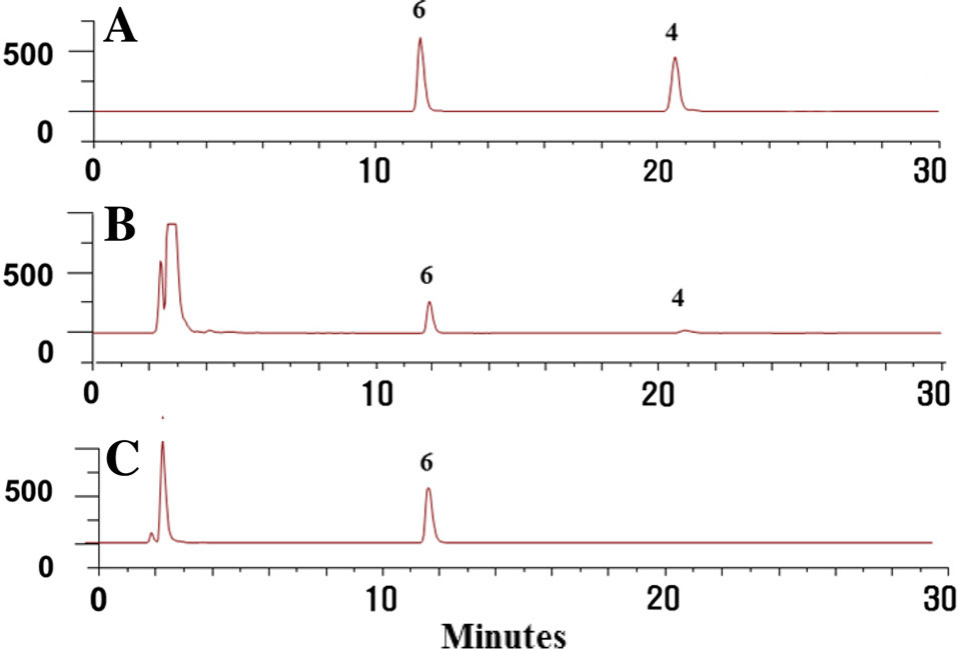
HPLC–ELSD chromatograms of standard mixture (**a**), the sample of *Z. jujuba* var. *spinosa* (J01; **b**) and the sample of *Z. mauritiana* (M01; **c**). *4* Jujuboside A, *6* Naringenin

HPLC conditions were selected to obtain good resolution on the chromatograms within a short retention time. We investigated YMC J’sphere ODS-H80, YoungJinBioChrom Aegispak C18-L, and Phenomenex Gemini ODS C18 columns to optimize the HPLC–UV chromatographic conditions. These three columns showed similar results, but ODS-H80 showed better resolution and theoretical plate of each peak in robustness. Above three columns also showed similar results for HPLC–ELSD, but Aegispak C18-L showed better resolution and theoretical plate for jujuboside A. UV detector was used for magnoflorine, spinosin and 6′′′-feruloyl spinosin because these compounds have good absorption in UV wavelengths. We used 270 nm because this was the maximum absorption of the three compounds. Mobile phase of water–methanol was adequate for good resolution of compounds during UV and ELSD. Adding 0.1 % formic acid to both water and methanol significantly improved the separation. Furthermore, we set the ELSD parameters for a spray chamber and drift tube temperatures, and gas pressure, with the purpose of generating a reproducible jujuboside A peak. Ultimately, the optimal mobile phase was a 0.1 % formic acid in methanol and a 0.1 % formic acid in deionized water in the gradient elution followed by the isocratic elution mode. Typical chromatograms of the sample and standard mixtures are shown in Figs. 2 and 3; the target compounds including I.S. were completely separated within 40 min by UV, and 30 min by ELSD. Naringin was selected as the I.S. for UV, and naringenin for ELSD (Fig. 1).

### Optimization of the sample preparation conditions

**Fig. 4:**
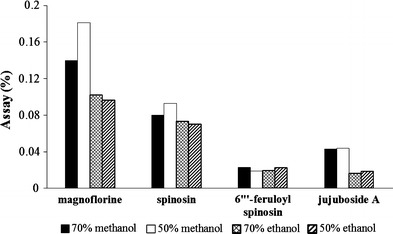
Comparison of the extraction solvents for extraction efficiencies of marker compounds (*n* = 3, w/w%)

**Fig. 5:**
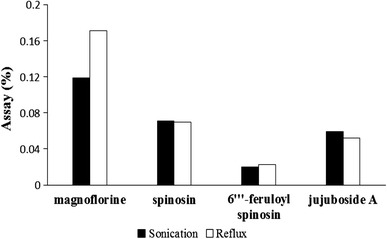
Comparison of the extraction methods (*sonication and reflux*) for extraction efficiencies of marker compounds (*n* = 3, w/w %)

**Fig. 6:**
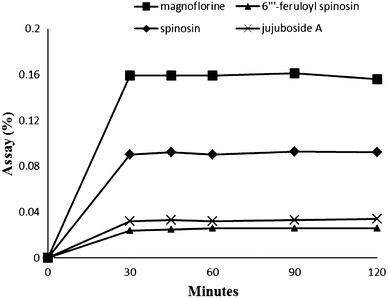
Comparison of the extraction time for extraction efficiencies of marker compounds (*1* = 3, w/w %)

Four extracting solvents, including 70 % ethanol, 50 % ethanol, 70 % methanol, and 50 % methanol containing 80 ppm of naringin (I.S.) for HPLC–UV and containing 50 ppm of naringenin (I.S.) for HPLC–ELSD, were compared in sample assays after extraction by sonication for 30 min at room temperature. When the samples were extracted with 50 % methanol, the sample assays were higher than the other solvent samples in both methods. Therefore, we employed 50 % methanol as the extracting solvent throughout this work (Fig. 4). Ultra-sonication and reflux using each 50 % methanol extraction solvent containing 80 ppm of naringin (I.S.) for HPLC–UV and containing 50 ppm of naringenin (I.S.) for HPLC–ELSD were compared as extraction methods in sample assays. Extraction by reflux showed better results than extraction by sonication for HPLC–UV. However, extraction by sonication showed better results than extraction by reflux for HPLC–ELSD (Fig. 5). To determine the time needed to complete the extraction, samples were extracted for 30, 45, 60, 90 and 120 min. When the extraction time was set to 30 min, the sample assay results were similar to those of the others in both methods. Therefore, all of the compounds were sufficiently extracted when the extraction time was 30 min (Fig. 6). The stability of naringin was compared between standing at room temperature and reflux at 80 °C for 30 min in 50 % methanol.

### Linearity, calibration range, and limits of detection and quantification

The calibration curves showed good linearity (*r* ^2^ > 0.999) within the test ranges, as shown in Table 1. The stock solution containing the reference compound was diluted with methanol to give a series of appropriate concentrations and the aliquots of the diluted solutions were injected. The LOD (S/N = 3) and LOQ (S/N = 10) values for magnoflorine, spinosin, 6′′′-feruloyl spinosin, and jujuboside A are presented in Table 1. The values for both LOD and LOQ for these four standards were low enough to detect traces of these compounds in either a crude extract or its preparation.

**Table 1:** Linearity, linear ranges, LOD and LOQ

Analytes	Regression equation	Linearity range (μg/mL)	Correlation coefficient (*r* ^2^)	LOD (μg/mL)	LOQ (μg/mL)
Magnoflorine^a^	0.0231x + 0.0361	0.625–200	0.9999	0.0072	0.0541
Spinosin^a^	0.0146x + 0.0441	0.625–200	0.9997	0.0135	0.0451
6′′′-Feruloyl spinosin^a^	0.0183x + 0.0283	0.625–200	0.9991	0.0158	0.0528
Jujuboside A^b^	1.7520x − 3.3979^c^	25–200	0.9990	12.565	41.884

^a^ HPLC–UV data

^b^ HPLC–ELSD data

^c^ In the regression equation of HPLC–ELSD, y = ax + b; y and x are the logarithmic values of peak area and concentration (μg/mL) of the marker compounds, respectively

### Precision and accuracy

The extraction precision and accuracy were assessed by extracting a known amount of compounds from Zizyphi Semen powdered samples. Known amounts of each standard compound at three levels were mixed with the sample powder and then extracted with 50 % methanol. Average recovery was calculated by the formula: *R* (%) = [(amount from the sample spiked standard − amount from the sample)/amount from the spiked standard] × 100. Intra-assay precision and accuracy were determined from the variability obtained from multiple analyses (*n* = 5) of quality control samples analyzed within the same analytical run. The quality control samples had intra-assay precision ≤4.82 % and accuracy of 95.18–101.37 %. Inter-assay precision and accuracy were evaluated from the differences in multiple analyses (*n* = 3) of quality control samples analyzed for 3 consecutive days. The quality control samples had an inter-assay precision of ≤3.17 % and accuracy of 97.61–101.87 %. Thus, the methods were highly reproducible. The precision and accuracy data are presented in Table 2.

**Table 2:** Precision and accuracy of analytical results

Analyte	Fortified conc. (μg/mL)	Sample conc. (μg/mL)	Intra-day (*n* = 5)				Sample conc. (μg/mL)	Inter-day (*n* = 3)			
			Observed (μg/mL)	SD	Accuracy (%)	Precision (%)		Observed (μg/mL)	SD	Accuracy (%)	Precision (%)
Magnoflorine^a^	0.9	13.28	14.18	4.82	100.06	4.78	13.32	14.24	3.23	101.87	3.17
	90.0	13.23	102.81	3.61	99.65	3.63	13.20	103.34	0.32	101.48	0.32
	135.0	13.14	150.00	2.87	101.37	2.83	13.16	150.92	0.56	100.27	0.56
Spinosin^a^	0.9	16.70	17.58	2.33	97.70	2.91	16.21	17.09	0.59	98.29	0.60
	90.0	16.98	104.82	2.75	97.61	2.82	16.46	104.30	0.29	99.06	0.29
	135.0	16.84	147.27	2.81	96.61	2.41	16.52	152.49	1.29	100.94	1.28
6′′′-Feruloyl spinosin^a^	1.0	4.56	5.55	1.95	99.72	1.96	4.17	5.16	1.44	98.41	1.46
	100.0	4.54	102.31	3.46	97.78	3.53	4.29	102.25	0.70	99.77	0.70
	150.0	4.54	155.29	1.06	100.50	1.06	4.28	154.38	0.75	99.46	0.76
Jujuboside A^b^	40.0	61.26	99.33	0.36	95.18	0.38	59.40	98.44	1.44	97.61	1.47
	100.0	61.26	161.60	3.74	100.34	3.73	59.40	160.77	0.46	101.38	0.45
	200.0	61.26	259.26	4.76	99.00	4.81	59.40	257.82	0.46	99.21	0.47

^a^ HPLC–UV data

^b^ HPLC–ELSD data

### Robustness

Robustness was determined to evaluate the reliability of the established HPLC method. The experimental conditions, such as column temperature, column species and flow rates, were purposely altered, and the theoretical plate (*N*), retention factor (*k*), separation factor (*α*) and resolution (*Rs*) were evaluated. The four analytical factors showed that the experimental conditions were sufficiently robust (data not shown).

### Sample analysis

The HPLC method was applied to analyze 91 samples corresponding to the seeds of 48 *Z. jujuba* var*. spinosa* (J01–J48) and 43 *Z. mauritiana* (M01–M43) samples. The average contents (wt%) of magnoflorine, spinosin, 6′′′-feruloyl spinosin, and jujuboside A are presented in Table 3. The average content of magnoflorine (0.156 %) in the *Z. jujuba* var*. spinosa* samples was higher than that of *Z. mauritiana* (0.055 %). In contrast, the average contents of spinosin (0.104 %) and 6′′′-feruloyl spinosin (0.040 %) in the *Z. jujuba* var*. spinosa* samples was lower than those of spinosin (0.142 %) and 6′′′-feruloyl spinosin (0.052 %) in *Z. mauritiana*. Interestingly, the average content of jujuboside A in the *Z. jujuba* var*. spinosa* samples was 0.058 %, whereas there was no jujuboside A in *Z. mauritiana*.

**Table 3:** Average contents (wt%) of magnoflorine, spinosin, 6′′′-feruloyl spinosin, and jujuboside A in Zizyphi Semen

	Mean ± SD (wt%)			
	Magnoflorine	Spinosin	6′′′-Feruloyl spinisin	Jujuboside A
*Z. jujuba* var*. spinosa* (*n* = 48)	0.1560 ± 0.0338	0.1042 ± 0.0245	0.0395 ± 0.0135	0.0581 ± 0.0141
*Z. mauritiana* (*n* = 43)	0.0553 ± 0.0163	0.1419 ± 0.0473	0.0516 ± 0.0160	N/D

Each value represents the mean ± SD (*n* = 3)

*N/D* not detected

This quantitative analysis results of magnoflorine, spinosin, 6′′′-feruloyl spinosin, and jujuboside A will be reflected in the contents regulation of these four compounds for Zizyphi Semen in the next revision of the K.P.

### Pattern recognition analysis

**Fig. 7:**
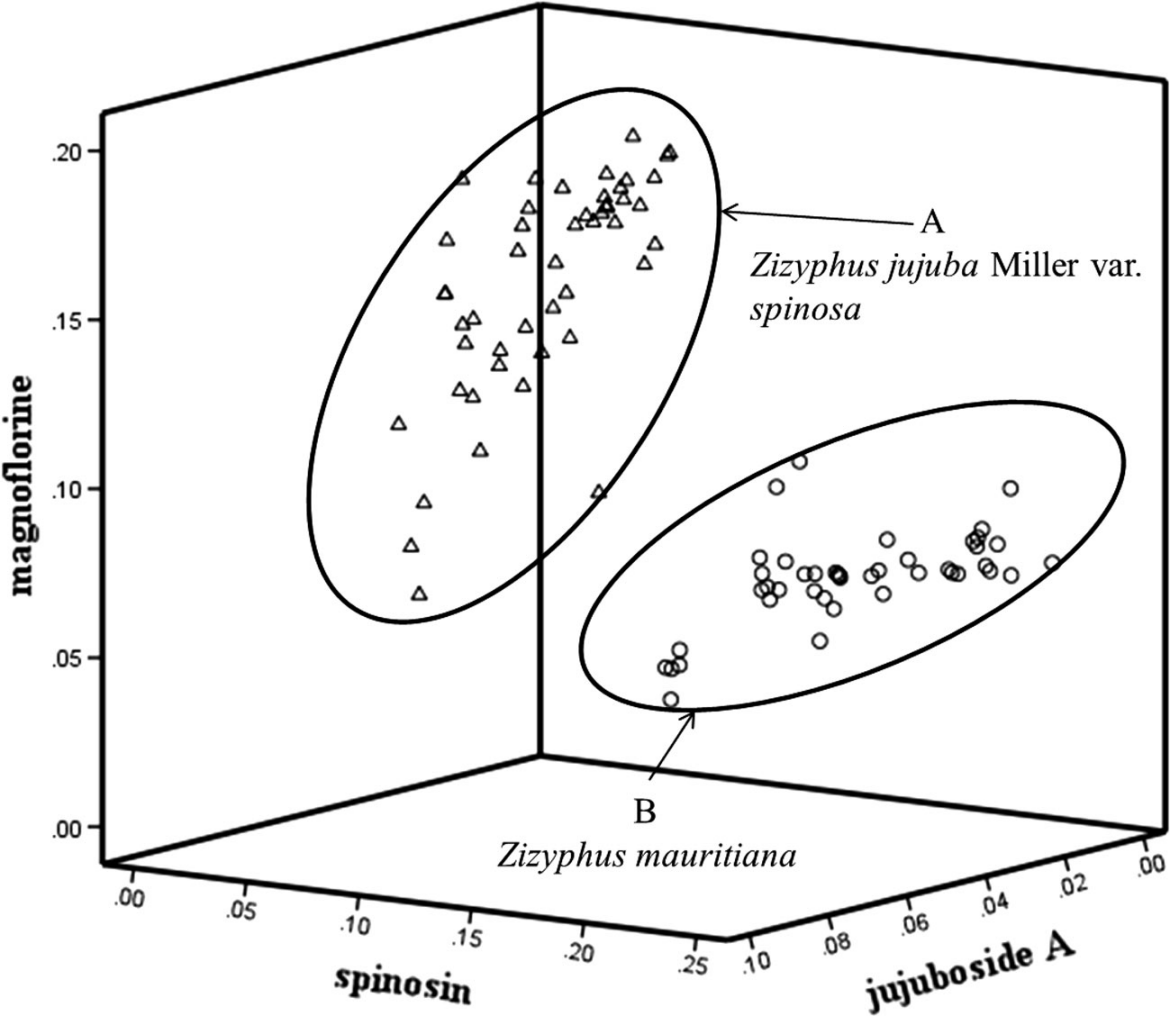
PAM of 91 authentic specimens. Group A (*open triangle*): *Z. jujuba* var*. spinosa*, Group B (*open circle*): *Z. mauritiana*

To evaluate the phytochemical equivalency among the seeds of the 48 *Z. jujuba* var*. spinosa* (J01–J48) and 43 *Z. mauritiana* (M01–M43) samples, pattern recognition analysis was conducted using the contents of three (magnoflorine, spinosin, and jujuboside A) and four (magnoflorine, spinosin, 6′′′-feruloyl spinosin, and jujuboside A) marker compounds. The content of 6′′′-feruloyl spinosin did not affect the result of the pattern recognition, because the average 6′′′-feruloyl spinosin content between *Z. jujuba* var*. spinosa* and *Z. mauritiana* was not much different compared to that of the other three marker compounds. Therefore pattern recognition analysis was conducted using the magnoflorine, spinosin and jujuboside A contents. Consequently, considering the concatenation of the three compounds which was significantly different between two species of *Z. jujuba* var*. spinosa* and *Z. mauritiana,* all of the samples were divided into two groups, *Z. jujuba* var*. spinosa* (A) and *Z. mauritiana* (B), by the pattern analysis (Fig. 7).

## Discussion

We have provided a fully validated HPLC method for quality control of Zizyphi Semen and pattern recognition analysis resulted in distinguishing between *Z. jujuba* var*. spinosa* and *Z. mauritiana*. The analytical conditions using a simple gradient elution system with UV and ELSD detectors allowed for a concise experiment and enhanced the analytical conditions. Our results suggest that magnoflorine, spinosin and jujuboside A are marker compounds for quality evaluations of Zizyphi Semen. Magnoflorine was not adopted as a marker compound of Zizyphi Semen in the C.P., even though the magnoflorine content was higher than that of spinosin from our assay results. Consequently, we suggest that including magnoflorine together with spinosin and jujuboside A as marker compounds is more reasonable compared with the marker compounds (spinosin and jujuboside A) currently in the C.P.
